# Effects of obesity, metabolic syndrome, and non‐alcoholic or alcoholic elevated liver enzymes on incidence of diabetes following lifestyle intervention: A subanalysis of the J‐DOIT1

**DOI:** 10.1002/1348-9585.12109

**Published:** 2020-01-21

**Authors:** Naoki Sakane, Kazuhiko Kotani, Akiko Suganuma, Kaoru Takahashi, Juichi Sato, Sadao Suzuki, Kazuo Izumi, Masayuki Kato, Mitsuhiko Noda, Shinsuke Nirengi, Hideshi Kuzuya

**Affiliations:** ^1^ Division of Preventive Medicine Clinical Research Institute National Hospital Organization Kyoto Medical Center Kyoto Japan; ^2^ Divison of Community and Family Medicine Jichi Medical University Tochigi Japan; ^3^ Hyogo Health Service Association Hyogo Japan; ^4^ Department of General Medicine/Family & Community Medicine Nagoya University Graduate School of Medicine Nagoya Japan; ^5^ Department of Public Health Nagoya City University Graduate School of Medical Sciences Nagoya Japan; ^6^ National Center for Global Health and Medicine Tokyo Japan; ^7^ Health Management Center and Diagnostic Imaging Center Toranomon Hospital Tokyo Japan; ^8^ Ichikawa Hospital International University of Health and Welfare Chiba Japan; ^9^ Koseikai Takeda Hospital Kyoto Japan

**Keywords:** diabetes prevention, impaired fasting glucose, non‐alcoholic fatty liver disease

## Abstract

**Objectives:**

Using annual health check‐up data, the aim of this study was to identify target populations for lifestyle interventions to effectively prevent diabetes in a real‐world setting.

**Methods:**

The Japan Diabetes Outcome Intervention Trial‐1, a prospective, cluster‐randomized controlled trial, was launched to test if year‐long telephone‐delivered lifestyle support by health professionals can prevent the development of type 2 diabetes (T2D) in people with impaired fasting glucose (IFG) identified at health check‐ups. A total of 2607 participants aged 20‐65 years with IFG were randomized to an intervention arm (n = 1240) or a control arm (n = 1367). We performed subgroup analysis to examine the effects of the intervention on the incidence of T2D in participants with body mass index (BMI) ≥25, metabolic syndrome (MetS), and non‐alcoholic or alcoholic elevated liver enzymes at the baseline. Cox regression analysis adjusted for sex was used to calculate the hazard ratios (HRs).

**Results:**

In addition to IFG, the presence of BMI ≥25, MetS, and elevated liver enzymes increased the incidence of diabetes by two‐ or three‐fold. During a median follow‐up period of 4.9 years, only the non‐alcoholic elevated liver enzyme group showed a low incidence rate owing to lifestyle interventions (adjusted HR: 0.42, 95% confidence interval: 0.18‐0.98).

**Conclusion:**

The results suggest that people who have IFG and non‐alcoholic elevated liver enzymes are a good target population for lifestyle interventions to effectively reduce the incidence of diabetes in a real‐world setting.

## 1 INTRODUCTION

With type 2 diabetes (T2D) associated with increased risks of morbidity and mortality, diabetes prevention is an urgent global issue.^1^ In Japan, it is mandatory for all adults to undergo annual health check‐ups.^2^, ^3^ Utilizing these health check‐ups effectively may be the key to diabetes prevention at the national level.

The Diabetes Prevention Program (DPP) and the Finnish Diabetes Prevention Study revealed that intensive lifestyle interventions reduced the incidence of T2D in high‐risk populations with obesity.^4^, ^5^ However, given the limited resources for primary healthcare, research is required to establish strategies to maximize cost effectiveness.^6^, ^7^ In existing studies, the target populations have mainly included individuals with obesity with or without prediabetes, and the main outcome reported in all studies has been weight change.^1^ For example, the SHINE study, using phone call‐delivered lifestyle interventions based on the DPP, targeted people with obesity who had metabolic syndrome (MetS), and the main outcome was weight change.^8^ Weight reduction was achieved through workplace intervention in the recent peer‐reviewed literature, though such interventions varied substantially in their effectiveness. 9

We launched the Japan Diabetes Outcome Intervention Trial‐1 (J‐DOIT1) to test whether phone call‐delivered lifestyle support by health professionals could reduce the incidence of T2D in participants with impaired fasting glucose (IFG) identified during health check‐ups.^10^ The body mass index (BMI) distribution in J‐DOIT1 participants ranged widely from lean, to normal, to obesity. It is well known that non‐alcoholic fatty liver disease (NAFLD) is associated with an increased risk of diabetes.^11^, ^12^, ^13^ However, it is unclear whether people with NAFLD are an effective target population for DPPs. To assess the liver condition, we had to rely on data on liver enzymes, because annual health check‐ups do not include abdominal ultrasonography. We adopted the definition of NAFLD based on the population‐based FIN‐D2D survey.^14^

The aim of the study was to examine the comparative effects of obesity, MetS, and non‐alcoholic or alcoholic elevated liver enzymes on the incidence of T2D following lifestyle intervention in a real‐world setting.

## 2 MATERIALS AND METHODS

### 2.1 Ethics statement

This study was approved by the Ethical Committee of the Japan Foundation for the Promotion of International Medical Research Cooperation (H181211).

This study was conducted according to the principles of the Declaration of Helsinki. This trial has been registered with the University Hospital Medical Information Network (UMIN000000662).

### 2.2 Study design

The J‐DOIT1 is a two‐armed cluster randomized controlled trial with randomization at the level of the healthcare division with a follow‐up period of 5.5 years. Healthcare divisions in companies and communities practicing health check‐up services formed a cluster and participated in the J‐DOIT1. Participants in the intervention arm received year‐long phone call‐delivered lifestyle support from healthcare professionals while participants in the control arm did not. A weight scale and a pedometer with a storage function were provided to all participants. A detailed description of the design has been published elsewhere.^10^ The groups of healthcare divisions were randomly assigned to the intervention or control arm.

### 2.3 Participants

Participants aged 20‐65 years with IFG—defined as a fasting plasma glucose (FPG) concentration of 100‐125 mg/dL (5.6‐5.9 mmol/L)—were included in our study. Individuals already diagnosed with diabetes, with a history of administration of anti‐diabetic agents, and an HbA1c of ≥6.5% were excluded. We also excluded individuals with medical conditions that preclude exercise, type 1 diabetes mellitus, pregnancy, liver cirrhosis, or chronic viral hepatitis, and those with a cardiac pacemaker device.

### 2.4 Definitions

Obesity was defined as BMI ≥25 kg/m^2^ according to the WHO Western Pacific Regional Office criteria.^15^ MetS was defined on the basis of modified criteria from the third report of the NCEP/ATP III.^16^, ^17^ An individual was judged to have MetS on the basis of the presence of three or more of the following components: (a) serum triglycerides ≥150 mg/dL, (b) high‐density lipoprotein (HDL) cholesterol <40 mg/dL for men and <50 mg/dL for women, (c) FPG ≥ 100 mg/dl, (d) blood pressure ≥130/85 mmHg or the use of blood pressure‐reducing agents, (e) BMI ≥25 kg/m^2^. In 2006, when baseline data were obtained, waist circumference (WC) was not measured at the majority of health check‐up sites. Therefore, BMI was used as a substitute for WC. This BMI level reportedly corresponds well to the Asian criterion for a large WC of ≥90 cm for men and ≥80 cm for women. 18 We adopted the definition of non‐alcoholic or alcoholic elevated liver enzymes based on the population‐based FIN‐D2D survey,^14^ because abdominal ultrasound examinations were not included in the annual health check‐ups. Increased liver fat content was defined as liver fat >5.6% based on the Dallas Heart Study, corresponding to aspartate aminotransferase (AST) levels of 33 and 29 U/L in men and women, respectively, and to alanine aminotransferase (ALT) levels of 43 and 30 U/L in men and women, respectively. In this study, men and women with increased ALT and/or AST levels consuming ≤20 g (for men) and ≤10 g (for women) of ethanol per day were considered to have non‐alcoholic elevated liver enzymes,^14^ while those consuming >20 g (for men) and >10 g (for women) of ethanol per day were considered to have alcoholic elevated liver enzymes.

### 2.5 Intervention

Participants in the intervention arm received telephone‐delivered lifestyle support. The goals for each participant were set on the basis of the following four points: 1) exercise habits (≥10 000 steps per day), 2) an appropriate body weight (5% weight loss in obesity), 3) dietary fiber intake, and 4) moderate alcohol consumption. After the goals were set, all participants received a pedometer and a weight scale. Based on these results, participants in the intervention arm received year‐long telephone‐delivered lifestyle support. As the primary outcome, incident diabetes was evaluated during the study period.

### 2.6 Outcome

The data were collected from annual health check‐ups. The development of diabetes was defined as (a) FPG ≥ 126 mg/dL (7.0 mmol/L) and (b) a diagnosis of diabetes or use of anti‐diabetic drugs. We extracted information on age, sex, weight, BMI, blood pressure, FPG, total cholesterol, HDL cholesterol, triglycerides, AST, ALT, and gamma‐glutamyltransferase from the annual health check‐up dataset.

### 2.7 Sample size, randomization, and blinding

Calculated on the assumption that the diabetes incidence is 4% per year and that intervention reduces the incidence by 50%, N would be 1100 with an alpha of 5% and power of 90%. When the intraclass correlation coefficients and cluster size were assumed to be 0.02 and 60, S and the number of clusters were 2398 and 40, respectively. However, the sample size in this study was not determined because of subanalysis of the J‐DOIT1. The groups of healthcare divisions were the randomization units. Groups were then randomly assigned to the intervention or control arm. Study participants and staff members were not blinded to the study arm status.

### 2.8 Statistical analysis

The analyses were conducted on an intention‐to‐treat basis, using Stata 13.1 (StataCorp) and SPSS 24.0 (SPSS Inc). Analysis was performed separately in the groups of participants with obesity, MetS, and elevated liver enzyme levels. We plotted cumulative Kaplan‐Meier curves for T2D development during follow‐up according to the obesity, MetS, and liver condition categories. We took into account the clustering effect in the main outcome analysis and subanalysis using the LWA model.^19^ Cox regression analysis adjusted for sex was used to calculate the hazard ratios (HRs) and 95% confidence intervals (CIs).

## 3 RESULTS

### 3.1 Participant flow, recruitment, and baseline data

We invited eligible individuals in each cluster to participate in the study. Approximately 20% of the individuals consented to participate. Finally, 2607 persons with IFG were enrolled: 1240 in the intervention arm and 1367 in the control arm. The median age of the participants was 49 years and 83.4% were men. The BMI ranged widely from <18.5 to >30 with a median of 24.3 kg/m^2^. The prevalence of obesity and MetS was 37.5% and 38.1%, respectively. The prevalence of participants with non‐alcoholic and alcoholic elevated liver enzymes was 7.1 and 13.8%, respectively. All participants were classified according to the presence or absence of obesity (BMI ≥25), presence or absence of MetS, and presence or absence of elevated liver enzymes. There were no differences between the intervention and control arms in terms of age, sex ratio, BMI, and clinical parameters at the baseline (Table 1). The intervention arm received five to six phone calls in a year.

**Table 1:** Baseline data in the control and intervention arms in each category

Variables	BMI category		Mets category		Liver condition category		
	≥25	<25	MetS	Non‐MetS	Elevated liver enzymes		Normal
					Non‐alcoholic	Alcoholic	
Number							
Intervention arm	475	765	463	753	97	168	975
Control arm	504	863	490	844	99	191	1077
Age, years							
Intervention arm	48.5 ± 7.4	49.1 ± 8	48.9 ± 7.4	48.8 ± 8.1	49.0 ± 8.7	47.8 ± 7.2	49.1 ± 7.8
Control arm	49.1 ± 7.1	48.7 ± 7.8	49.4 ± 6.7	48.4 ± 7.9	47.5 ± 8.2	49.1 ± 6.6	49.0 ± 7.6
Male, %							
Intervention arm	86.5	80.0	89.2	78.6	71.1	91.1	92.2
Control arm	87.7	82.0	90.0	80.9	76.8	92.7	83.3
BMI, kg/m^2^							
Intervention arm	27.6 ± 2.2	22.4 ± 1.9	26.5 ± 2.9	23.1 ± 2.6	26.4 ± 3.4	26.1 ± 3.1	23.9 ± 3.0
Control arm	27.4 ± 2.3	22.4 ± 1.8	26.6 ± 2.9	22.9 ± 2.2	26.4 ± 3.7	25.9 ± 3.2	23.8 ± 2.8
Systolic blood pressure, mmHg							
Intervention arm	129 ± 14.4	124.1 ± 15.8	133.2 ± 14.3	121.4 ± 14.2	126.3 ± 15.5	128.9 ± 13.1	125.4 ± 15.7
Control arm	130 ± 15.3	123.2 ± 15.7	134.1 ± 14.5	120.8 ± 14.4	126.3 ± 15.6	132.4 ± 14.9	124.5 ± 15.8
Diastolic blood pressure, mmHg							
Intervention arm	82.1 ± 10.7	77.4 ± 10.7	84.9 ± 10.3	75.6 ± 9.7	80.5 ± 11.6	81.5 ± 10.2	78.7 ± 10.9
Control arm	82.3 ± 10.7	77.5 ± 10.8	85.0 ± 10.5	76.1 ± 9.9	80.2 ± 11.5	83.7 ± 10.1	78.4 ± 10.9
Fasting plasma glucose, mmol/L							
Intervention arm	5.9 ± 0.3	5.9 ± 0.3	6.0 ± 0.3	5.9 ± 0.3	5.9 ± 0.4	6.0 ± 0.3	5.9 ± 0.3
Control arm	5.9 ± 0.3	5.9 ± 0.3	5.9 ± 0.3	5.9 ± 0.3	5.9 ± 0.3	6.0 ± 0.3	5.9 ± 0.3
HDL‐cholesterol, mmol/L							
Intervention arm	1.4 ± 0.3	1.6 ± 0.4	1.4 ± 0.3	1.7 ± 0.4	1.3 ± 0.3	1.5 ± 0.3	1.6 ± 0.4
Control arm	1.4 ± 0.3	1.7 ± 0.4	1.3 ± 0.3	1.7 ± 0.4	1.3 ± 0.4	1.5 ± 0.4	1.6 ± 0.4
Triglycerides, mmol/L							
Intervention arm	1.8 ± 1.4	1.4 ± 1.1	2.2 ± 1.4	1.1 ± 0.6	1.9 ± 1.3	2.0 ± 1.7	1.4 ± 1.1
Control arm	1.9 ± 1.3	1.4 ± 1.5	2.4 ± 2.0	1.1 ± 0.6	2.0 ± 1.8	2.1 ± 1.8	1.4 ± 1.3
Asparate aminotransferase, U/L							
Intervention arm	28.5 ± 15.2	23.0 ± 8.6	28.5 ± 14.1	23.1 ± 9.9	37.8 ± 18.2	39.9 ± 18.7	21.3 ± 4.4
Control arm	27.3 ± 11.4	23.7 ± 13.6	27.9 ± 11.4	23.4 ± 13.8	39.7 ± 29.3	39.7 ± 15.9	21.1 ± 4.2
Alanine aminotransferase, U/L							
Intervention arm	38.6 ± 25.5	24.5 ± 13.1	38.0 ± 24.6	25.0 ± 14.7	59.8 ± 27.3	56.1 ± 25.6	22.4 ± 7.8
Control arm	37.1 ± 22.8	24.9 ± 14.9	37.2 ± 22.0	25.0 ± 15.8	61.0 ± 28.2	53.3 ± 22.4	22.3 ± 7.7

Note

Values are the mean ± standard deviation or percentage. Subjects were classified according to the presence or absence of obesity, MetS, or liver conditions.

Abbreviations: BMI, body mass index; HDL, high‐density lipoprotein; MetS, metabolic syndrome.

### 3.2 Effects of obesity, MetS, and non‐alcoholic and alcoholic elevated liver enzymes on diabetes incidence

Participants with obesity or MetS had an approximately two‐fold higher incidence of diabetes compared with participants without obesity or MetS (2.2 and 2.0 times, respectively). Similarly, participants with non‐alcoholic or alcoholic elevated liver enzymes showed a two‐ to three‐fold higher incidence compared with those with normal liver function (Table 2). Thus, it was shown that the presence of obesity, MetS, and non‐alcoholic or alcoholic elevated liver enzymes further increased the risk of T2D in participants identified as having IFG during health check‐ups.

**Table 2:** Hazards ratio for the development of diabetes mellitus in the intervention or control arms according to the BMI, MetS, and liver condition categories

Categories	Intervention arm			Control arm			Hazard ratio	*P*‐value
	Number of incident diabetes	Person‐year at risk	Hazard per 100 person‐year at risk	Number of incident diabetes	Person‐years at risk	Hazard per 100 person‐year at risk		
BMI category								
≥25 kg/m^2^	56	1943	2.9	72	2089	3.5	0.84 (0.54‐1.29)	.422
<25 kg/m^2^	59	3187	1.9	60	3746	1.6	1.19 (0.82‐1.34)	.372
MetS category								
MetS	69	2009	3.4	75	2235	3.4	1.04 (0.72‐1.51)	.840
Non‐MetS	52	3442	1.5	69	4050	1.7	0.93 (0.61‐1.42)	.732
Liver condition category								
Elevated liver enzymes								
Non‐alcoholic	8	390	2.1	19	391	4.9	0.42 (0.18‐0.98)	.045
Alcoholic	20	701	2.9	32	786	4.0	0.71 (0.40‐1.25)	.240
Normal liver enzymes	87	4036	2.2	81	4658	1.7	1.25 (0.90‐1.72)	.180

Note

Cox regression analysis adjusted for sex was used to calculate the hazard ratio and 95% confidence interval (CI).

The figure shows incidence rates per 100 person‐years and corresponding hazard ratios and confidence intervals for the effects of intervention compared with control on the conversion of impaired fasting glucose to diabetes.

Abbreviations: BMI: body mass index, MetS: metabolic syndrome.

**Figure 1:**
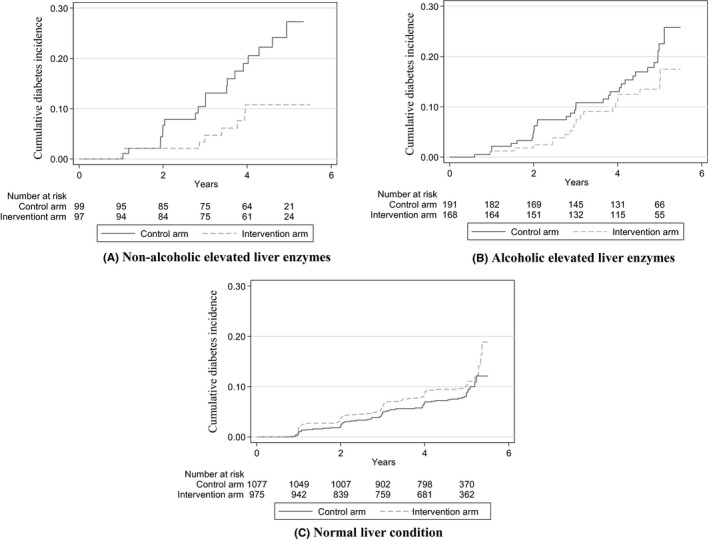
Cumulative incidence of type 2 diabetes according to the liver condition category. A, Non‐alcoholic elevated liver enzymes. B, Alcoholic elevated liver enzymes. C, Normal liver condition

No intervention effects were found in those with obesity or MetS (Table 2), while the intervention decreased the incidence of diabetes in participants with non‐alcoholic elevated liver enzymes (HR = 0.42, 95% CI = 0.18‐0.98) during a median follow‐up period of 4.9 years. No significant effects were noted in participants with alcoholic elevated liver enzymes. The cumulative incidence of T2D in participants with non‐alcoholic or alcoholic elevated liver enzymes and those with normal liver function is presented in Figure 1.

No serious adverse events related to the intervention were observed during the study.

## 4 DISCUSSION

The present study is the first randomized controlled trial to show that people with IFG and non‐alcoholic elevated liver enzymes may be a target population for diabetes prevention lifestyle interventions in a real‐world setting. NAFLD, the most prevalent chronic liver disease, is known to increase the risk of T2D.^11^, ^12^, ^13^ In the present subanalysis of the J‐DOIT1, we found that non‐alcoholic or alcoholic elevated liver enzymes were associated with a two‐ to three‐fold increased incidence of diabetes in participants with IFG. In the J‐DOIT1, when all participants with IFG were collectively analyzed, we could not show that our intervention method using phone calls was effective in controlling incident diabetes. The present subgroup analysis also failed to show that the intervention had effects on participants with MetS or obesity. Interestingly, with the same intervention, the development of diabetes was decreased in the non‐alcoholic elevated liver enzyme subgroup.

Non‐alcoholic fatty liver disease is the most common liver disorder in Western countries, affecting 17%‐46% of adults, with differences depending on the diagnostic method.^20^ Serum AST and ALT levels are said to be elevated in NAFLD, but as they are often within normal range in patients with NAFLD, they are considered poor markers.^21^, ^22^, ^23^ Actually, the prevalence of NAFLD (approximately 7%) determined in the present study using plasma aminotransferases was much lower than the prevalence determined using the gold standard of proton magnetic resonance spectroscopy (1H‐MRS) in the general population. This may be at least partially explained by the high cut‐off values we used for plasma aminotransferase levels. Thus, it is unlikely that all participants with NAFLD were identified in the present study. All stages of NAFLD could be present in participants without elevated enzymes. Therefore, we may not be able to extrapolate the present findings to all stages of NAFLD. However, we could, at least, argue that individuals identified as having IFG at health check‐ups should be given high priority for lifestyle modifications when they also show an unexplained elevation of liver enzymes. More sensitive serum biomarkers and scores are required for large epidemiological studies. In the present study, we could not determine the fatty liver index, conduct the SteatoTest, or calculate the NAFLD liver fat score,^24^, ^25^, ^26^ all of which are related to insulin resistance and known to reliably predict the presence of steatosis. However, as all available data for the present study were from annual health check‐ups and a self‐administered questionnaire on lifestyle, we could not perform any additional tests such as to determine serum insulin levels.

It has been proposed that NAFLD is a hepatic manifestation of MetS. There is a strong relationship between hepatic steatosis and insulin resistance. In people with NAFLD, excess fat deposition in the liver may lead to hepatic insulin resistance, resulting in an increase in hepatic glucose production. Lifestyle changes may cause a decrease in hepatic steatosis and an improvement in hepatic insulin resistance, reducing fasting glucose levels and preventing incident diabetes. A relatively small pool of intrahepatic lipids may be responsible for hepatic insulin resistance and increased rates of glucose production. Hepatic steatosis and hepatic insulin resistance would be reversed with modest weight reduction.

However, the present intervention did not reduce the incidence of T2D in participants with alcoholic elevated liver enzymes. The reason is not clear but reducing daily alcohol consumption is challenging; therefore, the present intervention did not affect the incidence of diabetes. Further efforts, including structured brief alcohol interventions for people with IFG and alcoholic fatty liver disease, are required.

## 5 STRENGTHS AND LIMITATIONS

The strengths of our study include the nationwide perspective, wide distribution of BMI, and real‐world setting. The limitations include single‐test results of FPG for identifying people with IFG. In addition, we did not adopt abdominal ultrasonography or liver biopsy, which does not accurately reflect the histopathology of NAFLD. However, although liver biopsy is the gold standard for NAFLD diagnosis, its use is limited because of the high cost, sampling errors, and procedure‐related morbidity and mortality.^27^ Although noninvasive techniques, such as ultrasound and computed tomography, are used for the diagnosis of hepatic steatosis in epidemiological studies, they are time‐consuming, require a well‐trained staff, and are costly; thus, they are not included in annual health check‐ups. Therefore, they cannot be used for NAFLD screening in a real‐world setting. There are many confounding factors, because this study was a subanalysis of the J‐DOIT1. The number of different techniques are described that may be applied to prevent or control for confounding: stratification and restriction.^28^ However, the number of events was limited in this study. Further examination including larger events are required to confirm these issues. Finally, owing to self‐selection bias or healthy volunteer bias, the generalizability of our findings may be limited, and extrapolating them to the general population may lead to an overestimation of the effects.

## 6 CONCLUSION

In conclusion, the presence of “unexplained” elevated liver enzymes may serve as a useful marker to identify individuals at risk of T2D in health check‐ups. This could be a good predictor of the effectiveness of lifestyle modifications. Thus, in primary healthcare settings, people with IFG and “unexplained” elevated liver enzymes should be prioritized for lifestyle interventions. Workplace interventions hold promise for preventing diabetes.^29^ More rigorous, creatively designed, workplace studies, are needed for employees at high risk for developing diabetes.

## DISCLOSURE

*Approval of the research protocol*: This study was approved by the Ethical Committee of the Japan Foundation for the Promotion of International Medical Research Cooperation (H181211). *Informed consent*: Written informed consent was obtained from all participants with full disclosure and explanation of the purpose and procedures of this study. *Registry and the registration no. of the study/trial*: This study was conducted according to the principles of the Declaration of Helsinki. This trial has been registered with the University Hospital Medical Information Network (UMIN000000662). *Animal studies*: N/A. *Conflict of interest*: None declared.

## AUTHOR CONTRIBUTIONS

NS, KI, NK, MN, and HK conceived the ideas. JS and SS curated data. AS, KT, JS, and SS analyzed the data. NS and HK acquired the funding. KI, MK, and MN collected the data. NS wrote the original draft. KK, SN, and HK wrote and reviewed the article.
